# An Evaluation of Nearly-Extinct Cohort Methods for Estimating the Very Elderly Populations of Australia and New Zealand

**DOI:** 10.1371/journal.pone.0123692

**Published:** 2015-04-07

**Authors:** Wilma Terblanche, Tom Wilson

**Affiliations:** 1 Queensland Centre for Population Research, School of Geography, Planning and Environmental Management, The University of Queensland, Brisbane, Queensland, Australia; 2 Advanced Demographic Modelling, New Farm, Brisbane, Queensland, Australia; London School of Economics, UNITED KINGDOM

## Abstract

The rapid growth of very elderly populations requires accurate population estimates up to the highest ages. However, it is recognised that estimates derived from census counts are often unreliable. Methods that make use of death data have not previously been evaluated for Australia and New Zealand. The aim was to evaluate a number of nearly-extinct cohort methods for producing very elderly population estimates by age and sex for Australia and New Zealand. The accuracy of official estimates was also assessed. Variants of three nearly-extinct cohort methods, the Survivor Ratio method, the Das Gupta method and a new method explicitly allowing for falling mortality over time, were evaluated by retrospective application over the period 1976-1996. Estimates by sex and single years of age were compared against numbers derived from the extinct cohort method. Errors were measured by the Weighted Mean Absolute Percentage Error. It is confirmed that for Australian females the Survivor Ratio method constrained to official estimates for ages 90+ performed well. However, for Australian males and both sexes in New Zealand, more accurate estimates were obtained by constraining the Survivor Ratio method to official estimates for ages 85+. Official estimates in Australia proved reasonably accurate for ages 90+ but at 100+ they varied significantly in accuracy from year to year. Estimates produced by Statistics New Zealand in aggregate for ages 90+ proved very accurate. We recommend the use of the Survivor Ratio method constrained to official estimates for ages 85+ to create very elderly population estimates for Australia and New Zealand.

## Introduction

Due mostly to substantial declines in older age mortality over the last few decades, Australia and New Zealand’s very elderly populations—defined here as those aged 85+—are growing faster than all younger age groups [1,2]. High birth rates up to the 1970s and continued improvements in survival to advanced ages lie behind projections of huge increases in very elderly numbers over the next few decades in both countries [3, 4]. The rapid growth of these very old populations has obvious implications for aged care, health care and social security benefits, thus creating a need for accurate and detailed population estimates [5,6].

However, official population estimates of the very elderly in Australia and New Zealand are affected by at least two long-standing limitations. The first relates to accuracy. Official population estimates for Australia, produced by the Australian Bureau of Statistics (ABS), and for New Zealand, produced by Statistics New Zealand, are based on census counts. It is widely recognised that at very high ages official population estimates and mortality rates suffer from inaccuracies originating from incorrect age information provided in census forms and errors introduced by census processing and editing [7–11]. The result is that at the highest ages population estimates are often inflated and mortality rates understated, with the degree of inaccuracy increasing with age [10, 12].

A second limitation of official population estimates is their publication by single years of age up to ages which are increasingly too low given the recent growth of very elderly populations. The ABS and Statistics New Zealand publish single year of age population estimates up to ages 99 and 89 respectively, with the final open-ended age groups being 100+ and 90+ [2, 13]. Rapidly growing numbers of centenarians and nonagenarians make single year of age mortality rates at these high ages increasingly important for robust projections of the very elderly.

Problems with very elderly population estimates are not unusual in countries without population registers. To overcome the shortcomings of census-based population figures, indirect estimation methods have been widely used to produce alternative estimates based on death data [8, 12, 14–16]. Because dates of birth are typically verified at death, ages on death certificates are generally regarded as more accurate than those given on census forms. Population estimates for the very elderly derived from death data are thus considered to be more reliable than official population estimates [11, 14]. Such estimates have been found to exhibit more plausible patterns over time and across ages, especially at ages above 90, and are very accurate when compared with estimates from population registers [17]. The use of death data to create population estimates also ensures consistency between numerator and denominator in the calculation of mortality rates, resulting in more accurate rates than those derived from deaths and census-based population estimates [12].

Two broad indirect estimation approaches using death data are commonly applied. The extinct cohort (or extinct generation) method is the simplest. It calculates historical population numbers for cohorts for which all members have died. A population estimate for any year and age of the cohort is obtained simply by summing all subsequent cohort deaths assuming that net migration is negligible [18]. However, the method must be adapted when a cohort is not fully extinct. In such circumstances various methods, collectively described as nearly-extinct cohort methods, must be employed. These either estimate the cohort population at the most recent date or do so indirectly by estimating future cohort deaths. A set of population estimates can then be obtained by summing deaths along the cohort as in the extinct cohort method. Nearly-extinct cohort methods proposed in the literature include:

- the Survivor Ratio method, which estimates a cohort’s population from the survivorship of earlier cohorts [12,19],
- Das Gupta’s method, based on estimating future cohort deaths [20],
- the method of Coale and Caselli [21], which estimates very elderly populations from death counts and estimated rates of change in death rates by age,
- the Mortality Decline method, which estimates the survivorship of a cohort allowing for age-specific mortality decline over time [22],
- Meslé and Vallin’s [23] approach which, similar to that of Das Gupta, estimates future deaths for the youngest cohort from ratios observed among older cohorts,
- the Das Gupta Advanced method which estimates future cohort deaths but allowing for mortality decline [24].

These methods are described later.

In many countries mortality rates at high ages are declining over time [9]. This can either be allowed for explicitly or indirectly by adjusting the resulting population estimates. For example, survival improvement is typically indirectly allowed for by increasing the estimated populations produced by the Survivor Ratio and Das Gupta methods by a factor such that the total estimated population at ages 90 and older equal the total of official estimates at the calculation date [25,26]. This also ensures a smooth transition into official population estimates. With the Mortality Decline and Das Gupta Advanced (DA) methods, variants of the Survivor Ratio and Das Gupta methods respectively, explicit allowance is made for declines in mortality rates by adjusting survivor ratios and death ratios. These methods are not dependent on the existence of reliable official population estimates for ages 90+ [24].

Over ten years ago, Thatcher, Kannisto and Andreev [25] published the results of an empirical comparison of several variants of the Survivor Ratio, Das Gupta and Mortality Decline methods applied to nine countries (Denmark, England & Wales, Finland, France, Japan, Netherlands, Norway, Sweden and Switzerland). The methods were applied retrospectively to estimate very elderly populations over the period 1960–95, and the results compared to estimates derived solely from the extinct cohort method. Based on average rankings of absolute deviations of estimated numbers from observed numbers at ages 95+ and ages 100+, they concluded that the Survivor Ratio method constrained to official 90+ population estimates produced the most accurate results. This method is now used for producing estimates in the Human Mortality Database (HMD) at ages 80 to 110+ for 37 countries, including Australia and New Zealand [26], and is also applied by the UK’s Office for National Statistics [27].

However, this constrained Survivor Ratio method is based on an assumption of reliable official population estimates for ages 90+. For situations where this is not the case, Andreev [24] proposed a modification of Das Gupta’s approach, termed the Das Gupta Advanced method. The current populations of nearly-extinct cohorts are found by estimating the likely number of future cohort deaths taking into account future mortality rate decline, and then summing those future deaths. Andreev applied the method to the US population with good results.

Both these studies made very important contributions to the literature. However, they are now more than ten years old, and very elderly population numbers have grown substantially since then, with the possibility that the best method may have changed over time. Moreover, neither study included Australia or New Zealand. The aim of this paper, therefore, is to retrospectively test a number of nearly-extinct cohort methods for estimating very elderly populations in Australia and New Zealand in order to determine which is most accurate. We investigate which combinations of parameters produce the lowest errors. These parameters relate to the age range considered when deriving the proportion of a cohort that is still alive, and the number of older cohorts averaged. We also explore whether results constrained to official estimates are more accurate than those without constraining, and whether it is best to explicitly allow for mortality decline over time or just take it into account implicitly. We additionally assess the accuracy of official very elderly population estimates.

The rest of the paper is organised as follows. The nearly-extinct cohort methods used to derive population estimates from death counts are set out in the next section. We then present the results of the retrospective indirect estimation, comparing estimates to population numbers obtained from the extinct cohort method alone. We conclude with a summary and recommendations.

## Data and Methods

The nearly-extinct cohort methods assessed for this paper were the Survivor Ratio and Das Gupta methods together with a new technique adapted from the Das Gupta Advanced and Mortality Decline methods, termed here the Survivor Ratio Advanced method. They are all described below. The accuracy of these non-extinct cohort methods was assessed by retrospectively applying them to create estimates for 31st December each year from 1976 to 1996, and comparing the results against more robust estimates derived from the extinct cohort method.

### Data

Deaths for Australia and New Zealand by sex and single years of age (age last birthday at date of death) up to 109 for 1960 to 2009 (Australia) and 2008 (New Zealand) were downloaded from the Human Mortality Database [28]. Deaths for Australia from 2010 to 2012 were obtained from the ABS [29]. No clear evidence of age heaping or overstatement were found in very elderly death data for Australia or New Zealand from 1965. Formal birth and death registration systems were implemented from 1838 to 1870 in the various Australian states and territories and from 1840 in New Zealand, and were thus in place when the cohorts considered in this study were born. Other studies also found Australia’s and New Zealand’s very elderly death data from 1971 to be of acceptable quality [30, 31].

Estimated Resident Populations (ERPs), the official population estimates in Australia and New Zealand, were required to apply 85+ and 90+ constraining to nearly-extinct cohort estimates, and to permit a comparison with extinct cohort estimates to be made. They were obtained by sex and single years of age from the ABS and Statistics New Zealand respectively [2, 13]. During 2013 the ABS changed the method of allowing for undercount when deriving ERPs from census counts and as a result, recalculated ERPs from 1991. The recalculated ERPs were used in this study. Because extinct cohort and nearly-extinct cohort methods were used to create 31st December estimates for each year, the 30th June ERPs were interpolated to 31st December.

### Population estimation methods

#### Extinct cohort method

The extinct cohort method estimates a cohort’s population for any year and age by summing subsequent cohort deaths [9, 18]. Algebraically, the population, P, of the cohort aged x last birthday on 31st December of year t for cohort c is:

$$P_{x,t}=\sum_{i=1}^{\omega -x}D_{t+i}^{c}$$ (1)

where ω is the age of extinction, and $D_{t+i}^{c}$ is the number of deaths in year t+i from cohort c, which are people born in the year t-x.

For this study, ω was estimated as the highest age at where there was expected to be only one survivor, consistent with the application by Thatcher, Kannisto and Andreev [25]. This age varied between years and between males and females but did not exceed 110. Hence, for Australia, the extinct cohort method was used to produce estimates for cohorts born before 1902, whilst for New Zealand it was applied to cohorts born before 1900 for females and 1902 for males. In fact, it was also used to create estimates for cohorts born up to 1906 by ‘completing’ the number of deaths in these very nearly extinct cohorts. There is therefore a small degree of indirect estimation in these extinct cohort estimates. However, given the tiny numbers involved, the possible extent of error is very small.

#### Survivor Ratio (SR) method

The Survivor Ratio (SR) method estimates the size of a nearly-extinct cohort for a recent date and age on the basis of the survivorship of older cohorts to the same age. The population is obtained via a survivor ratio, defined as the ratio of a cohort’s population at the calculation date to its size k years ago. It may be expressed as:

$$R_{x}^{c}=\frac{P_{x,t}^{c}}{P_{x-k,t-k}^{c}}$$ (2)

In line with the extinct cohort method, the number of survivors from a particular cohort k years earlier can be written as:

$$P_{x-k,t-k}=P_{x,t}+\sum_{i=0}^{k-1}D_{t-i}^{c}$$ (3)

so that the survivor ratio for this cohort over k years can be expressed as:

$$R_{x}=\frac{P_{x,t}}{P_{x,t}+\sum_{i=0}^{k-1}D_{t-i}^{c}}$$ (4)

The estimated population aged x at 31st December of year t can be obtained by solving for *P*_*x*,*t*_

$$P_{x,t}=\frac{R_{x}}{1-R_{x}}\sum_{i=0}^{k-1}D_{t-i}^{c}$$ (5)

Then the cohort’s population in earlier years and younger ages may be obtained by summing deaths as in the extinct cohort method. To smooth out random variations, the estimated survivor ratio in Eq 5 is usually based on the average experience of m older cohorts:

$$R_{x}=\frac{\sum_{j=1}^{m}P_{x,t-j}}{\sum_{j=1}^{m}P_{x-k,t-k-j}}$$ (6)

This method is denoted by SR(k, m, constraint), where k refers to the age range over which the survivor ratio is measured and m refers to the number of older cohorts over which the survivor ratio is averaged. ‘Constraint’ indicates whether a constraint was applied and, if so, the age range of the official population estimate. It is either ‘NC’, ‘85+’ or ‘90+’, referring to no constraint, constraining to the official 85+ population estimate, or the 90+ population estimate. Different variants assessed in this paper relate to different values for k, m and constraining population age groups.

#### Das Gupta’s (DG) method

Instead of starting from the assumed proportion of a cohort still alive at a certain date, Das Gupta’s method first estimates future cohort deaths, based on death ratios [22]. A death ratio is the number of deaths at a particular age relative to deaths at the previous age, averaged over a number of older cohorts. Future deaths that have not yet occurred among younger cohorts are then estimated by progressively applying these ratios at successive ages. Death ratios for each age are calculated at the end of the latest year for which death data are available, based on observed deaths by age for m older cohorts. Cohort population estimates are then derived in the same way as the extinct cohort method by summing cohort deaths (Eq 1).

The death ratio at age x, based on observed deaths among m older cohorts is calculated as:

$$dr_{x}=\frac{\sum_{j=1}^{m}D_{t-j}^{c-j}}{\sum_{j=1}^{m}D_{t-j-1}^{c-j}}$$ (7)

The numbers of deaths from a cohort at future dates and at higher ages are then derived by applying these death ratios successively:

$$D_{t}^{c}=dr_{x}\times D_{t-1}^{c}$$ (8)

A very similar approach was applied by Meslé and Vallin [23].

In the literature this method is denoted by DG(m, constraint), where m refers to the number of older cohort averaged and ‘constraint’ indicates whether constraining to official estimates for ages 85+ or 90+ is applied. However, a slightly different notation is used here. While Das Gupta’s method makes use of death ratios between consecutive ages, and the Survivor Ratio method makes use of survivor ratios measured over k years of age, it can be shown that the DG method produces exactly the same results as the SR method when k = 1. Therefore, while the calculations differ mechanically, method DG(m) gives the same results as SR(1, m), and the DG method can thus be considered a variant of the SR method. The algebraic proof of this is set out in S1 Appendix. Therefore, for consistency, the DG method will be denoted by DG(1, m, constraint) in this paper.

#### Methods estimating future deaths that explicitly allow for mortality decline

The Das Gupta Advanced (DA) method, introduced by Andreev [24], adjusts death ratios to reflect mortality decline over time prior to their application to derive estimated future cohort deaths. Similar to Coale and Caselli [21], these adjustments are based on an assumption that mortality decline diminishes linearly with increasing age. The validity of these methods relies on empirical evidence that the decline in mortality rates reduces with increasing age at ages 80 and above. This is not, however, evident in the Australian or New Zealand data. Rates of mortality decline increase with age in some years and are generally quite volatile. Overall, there are no clear and consistent patterns of decline with age.

The Mortality Decline (MD) method [22, 25] also allows for mortality decline over time. It involves modelling the temporal decline in mortality rates for a given age by fitting a loglinear function to mortality rates observed for a number of older cohorts. In this case the linear function is fitted over time rather than age. A new method, similar in concept to the MD method, but believed to be simpler, is evaluated in this paper. Similar to the MD method, it extrapolates average mortality decline observed in the previous ten cohorts to estimate the mortality for the youngest unobserved cohort. However, instead of fitting a loglinear curve to mortality rates at a particular age across ten older cohorts, the average annual improvement in survivor ratios is measured. The average improvement in survivor ratios measured at a specific age over ten older cohorts is assumed to apply to the youngest cohort. This method is referred to as the Survivor Ratio Advanced (SA) method, and is set out below.

According to Eq 2, the survivor ratio at a particular age for cohort c is calculated as:

$$R_{x}^{c}=\frac{P_{x,t}^{c}}{P_{x-k,t-k}^{c}}$$ (9)

The average improvement in the survivor ratios between subsequent cohorts can be written as:

$$r_{x}=\sum_{j=1}^{n-1}\left(\frac{R_{x}^{c-j}}{R_{x}^{c-j-1}}-1\right)/\left(n-1\right)$$ (10)

where n is the number of older cohorts for which changes in survivor ratios are measured. The survivor ratio based on m older cohorts (Eq 6) is then increased with this average improvement as follows:

$$R_{x}^{'}=\frac{\sum_{j=1}^{m}P_{x,t-j}}{\sum_{j=1}^{m}P_{x-k,t-k-j}}\times {\left(1+r_{x}\right)}^{\frac{1}{2}\left(m+1\right)}$$ (11)

This method is denoted by SA(k, m, NC, n) where k and m are as defined for the SR method.

Due to the volatility of survivor ratios at very high ages a combined improvement factor was calculated based on aggregate ratios for ages 103+ in the case of Australia and 100+ in the case of New Zealand. Care is needed where the rate of improvement is very high as this may result in survivor ratios of more than 1.

#### Variants tested in this paper

Table 1 lists the Survivor Ratio, Das Gupta and Survivor Ratio Advanced variants which were assessed.

**Table 1:** Almost-extinct cohort methods tested.

SR variants	DG variants	SA variants	Official estimates
SR(5,5,NC)	DG(1,5,NC)	SA(5,5,NC,10)	Estimated Resident Population (ERP)
SR(5,5,90+)	DG(1,5,90+)	SA(1,5,NC,10)	
SR(5,5,85+)	DG(1,5,85+)		
SR(5,3,NC)	DG(1,3,NC)		
SR(5,3,90+)	DG(1,3,90+)		
SR(5,3,85+)	DG(1,3,85+)		

Note. The notation is Method (age range, number of cohorts for averaging survivor ratio or death ratio, official population estimate used as a constraint). In the case of the SA method the last parameter refers to the number of older cohorts over which survivor ratio change is measured.

### Error Measures

Error is defined as the population estimate (E) minus the actual population (A), where ‘population estimate’ refers to numbers obtained by applying nearly-extinct cohort methods and ‘actual population’ describes the populations calculated by the extinct cohort method.

In order to compare the relative accuracy of different methods, Thatcher, Kannisto and Andreev [25] assigned ranks based on absolute errors for ages 95+ and 100+ in each year and for each country. Andreev [24] considered total relative errors above age 90. In focusing on relative errors, the extent of total deviations above certain ages may be understated if overestimations at some ages are offset by underestimations at others. When measuring deviation above certain ages, we believe it is more appropriate to measure absolute errors. Population numbers decrease rapidly with advancing age above 90. In order to measure the accuracy of estimation methods at ages 90 and higher or 100 and higher, it is therefore appropriate to apply a weight of population size at each individual age to the absolute error measured at that age, when determining the average. This measure of accuracy is referred to as the Weighted Mean Absolute Percentage Error (WMAPE) [32] and is calculated as follows:

$$WMAPE_{t}=\frac{\sum_{x}\frac{\left|E_{x}-A_{x}\right|}{A_{x}}\times 100\%\times A_{x}}{\sum_{x}A_{x}}$$ (12)

or simply

$$WMAPE_{t}=\frac{\sum_{x}\left|E_{x}-A_{x}\right|\times 100\%}{\sum_{x}A_{x}}$$ (13)

WMAPEs are calculated for each year and the summation is over ages 90 and older and ages 100 and older respectively. Lower WMAPEs indicate greater accuracy. The next section sets out the average of WMAPEs for each method over the whole period studied, followed by WMAPEs in individual years.

Because the highest ages at which ERPs are published are 100+ and 90+ for Australia and New Zealand respectively, a more appropriate measure for comparing the accuracy of methods at these ages is Percentage Error (PE). Percentage Error is calculated as follows:

$$PE_{t}=\frac{E-A}{A}\times 100\%$$ (14)

In order to compare the accuracy of the final open-ended age group ERPs with those of the other methods, Mean Absolute Percentage Error (MAPE) will be used, calculated as:

$$MAPE_{t}=\frac{\sum_{t}\left|PE_{t}\right|}{n}$$ (15)

where n refers to the number of years in the study.

## Results

### Best method on average over the 1976–96 period

#### Australia

**Fig 1:**
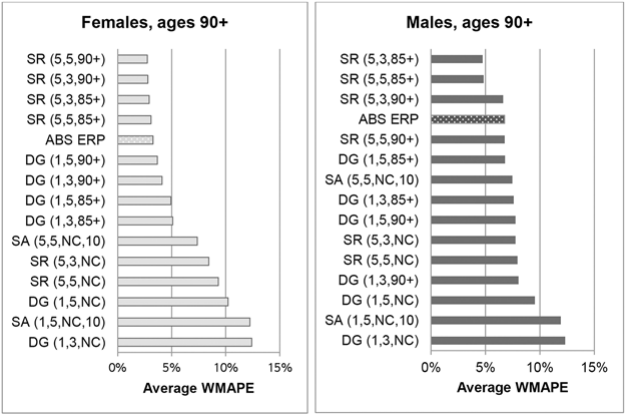
Weighted Mean Absolute Percentage Errors of population estimates for ages 90+ for Australia, averaged over 1976–1996.

**Fig 2:**
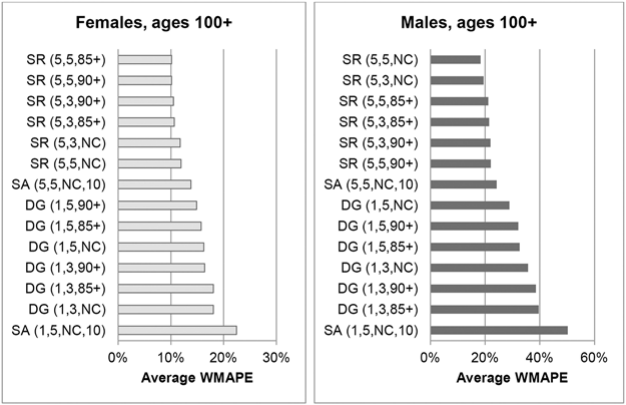
Weighted Mean Absolute Percentage Errors of population estimates for ages 100+ for Australia, averaged over 1976–1996. Note. ABS ERPs are not included in Fig 2 because only an aggregate ERP is available for ages 100+, which means WMAPE cannot be determined.

Figs 1 and 2 show the errors of each nearly-extinct cohort population estimation method at ages 90+ and ages 100+ for Australia. ERPs are not included in Fig 2 because only an aggregate 100+ ERP is available, which means WMAPE cannot be determined. MAPEs for ERPs at ages 100+ are discussed at the end of this section. The errors in Figs 1 and 2 are simple averages of WMAPEs for each year in the period 1976 to 1996. The graphs clearly demonstrate that errors tend to increase with age and are generally higher for males than females. The male populations aged 90+ were only around a quarter that of females and their numbers decrease rapidly with increasing age. The greater errors for males and at higher ages are partly due to increased volatility resulting from smaller volumes of data.

For females at ages 90+ the average WMAPE for the ABS ERPs proved relatively low at 3.3%. Not surprisingly therefore, variants where results were constrained to ABS 85+ or 90+ ERPs produced the best estimates, with WMAPEs varying between 2.7% and 5.1%. Results were not much different whether the 85+ or the 90+ ERP constraints were applied, but use of these constraints gave more accurate estimates than either explicitly allowing for an improvement in survivor rates or not constraining. Furthermore, variations in errors between different numbers of cohorts (m) were small, but errors were generally lower if based on longer age ranges (k). As shown in S1 Appendix, results from the DG method are equivalent to those from the SR method when k = 1. For the same number of cohorts, errors from the DG method were higher than for the SR method, indicating that the use of a 5-year age range yielded more accurate results than a one-year age range.

For populations aged 90+ average errors for the ABS ERPs for males (6.8%) were higher than those for females (3.3%). Preston, Elo and Stewart [10] found that the steeper the decline in population numbers with age, the greater the degree of overstatement of population estimates. The more rapid reduction in the number of males with increasing age compared to females could thus explain the greater error in ERPs for males. At 4.8% the SR variant SR(5,5,85+), constrained to the 85+ ERP, produced lower errors than SR(5,5,90+), constrained to the 90+ ERP (6.8%), the variant used in the HMD. An analysis of ERP errors for males at single ages from 85–99 shows the accuracy of ERPs deteriorates rapidly with increasing age from around age 93. This explains why, for males, constraining to 85+ ERPs resulted in more accurate estimates compared to constraining to 90+ ERPs. The accuracy of ERPs for females also deteriorates from around age 95 but to a much smaller extent than for males. Unconstrained variants of all methods also gave higher errors, and DG variants were also less accurate than SR variants.

For both males and females, the SA method based on a 5-year age range resulted in lower errors than unconstrained SR methods, as would be expected when mortality rates have been declining. Andreev [24] found that for the large countries in his study, relative errors for the DA method were lower than both unconstrained SR and DG variants, but results were less consistent for smaller populations. In this study it was found that the SA method based on survivor ratios measured at adjacent ages (k = 1) gave very poor results, as did the unconstrained DG method, which also used one-year age ranges. Lower errors were achieved when constraining to official estimates than explicitly allowing for survival improvement.

For female centenarians SR variants constrained to ABS 85+ or 90+ ERPs produced the lowest errors. Unconstrained SR variants were the most accurate for male centenarians. However, for both males and females, the differences in errors between constrained and unconstrained SR variants were small. For ages 100+, SR methods where survivor ratios were measured over 5-year age ranges gave the best results. This was the case whether based on the experience of 3 or 5 cohorts, and irrespective of whether estimates were constrained or not. The DG variants and the SA method based on 1-year age ranges proved to be the least accurate.

Table 2 shows the Mean Absolute Percentage Error (MAPE) for the methods tested for males and females aged 100+. Consistent with WMAPEs, SR(5,5,85+) generated the lowest MAPE for females at 9.2% and unconstrained SR variants generated the lowest MAPE for males at 12.8%. The accuracy of ABS ERPs for ages 100+ was relatively poor for both females and males with MAPEs of 16.1% and 34.4% respectively.

**Table 2:** Mean Absolute Percentage Error for males and females aged 100+ over 1976–1996.

	Females	Males
SR (5,5,85+)	9.2%	15.4%
SR (5,5,90+)	9.3%	15.9%
SR (5,3,90+)	9.5%	14.5%
SR (5,3,85+)	9.7%	14.7%
SR (5,3,NC)	10.0%	12.8%
SR (5,5,NC)	10.3%	12.8%
DG (1,5,90+)	12.2%	22.5%
SA (5,5,NC,10)	12.7%	17.8%
DG (1,5,NC)	12.8%	20.6%
DG (1,5,85+)	13.0%	22.9%
DG (1,3,90+)	13.5%	29.1%
DG (1,3,NC)	14.5%	27.1%
ABS ERP	16.1%	34.4%
SA (1,5,NC,10)	18.5%	42.2%
DG (1,3,85+)	19.3%	30.1%

#### New Zealand

**Fig 3:**
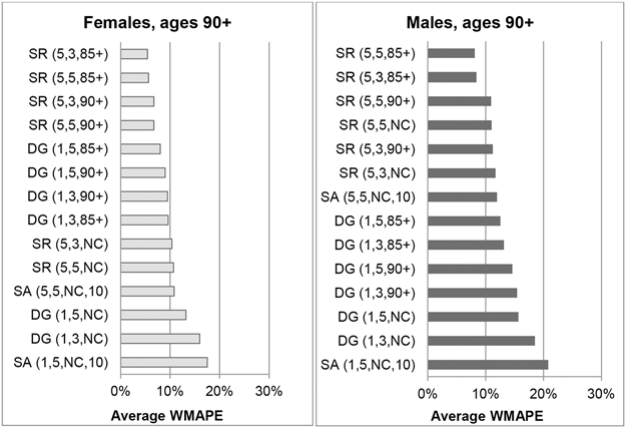
Weighted Mean Absolute Percentage Errors of population estimates for ages 90+ for New Zealand, averaged over 1976–1996.

**Fig 4:**
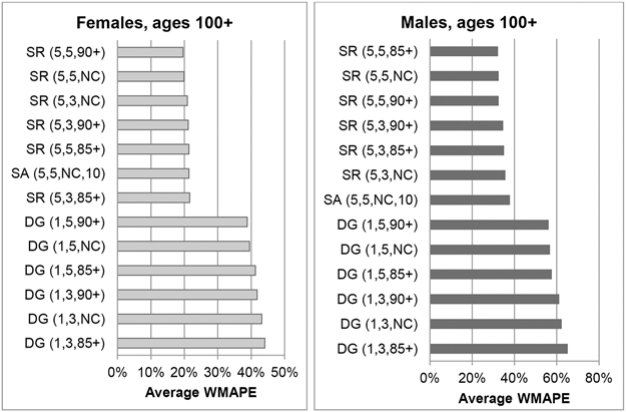
Weighted Mean Absolute Percentage Errors of population estimates for ages 100+ for New Zealand, averaged over 1976–1996. Note. Statistics New Zealand publishes only aggregate estimates for ages 90+. Therefore WMAPEs could not be calculated and are not shown.

Figs 3 and 4 show errors for each method for ages 90+ and 100+ for New Zealand, averaged over the period 1976 to 1996. ERPs are not shown because ERPs in New Zealand are only available up to age 90+ and WMAPEs can thus not be determined.

The population aged 90+ in New Zealand is around a fifth the size of that in Australia. At the end of 1996 there were almost 9,000 female nonagenarians in New Zealand, compared to around 14,000 male nonagenarians in Australia. If size and volatility were the main drivers, one could therefore expect errors of the nearly-extinct cohort estimates for the female population in New Zealand to be of similar size to those for Australian males. This does seem to be the case because errors for females aged 90+ in New Zealand varied from 4.6% to 17.5% compared to errors of 4.7% to 12.3% for Australian males aged 90+.

For ages 90+, SR variants constrained to ERPs for ages 85+ proved to be the most accurate, with average WMAPEs of 5.4% and 8.1% respectively for females and males. This was followed by SR variants constrained to ERPs for ages 90+, with WMAPEs of 6.7% for females and 10.9% for males. As was the case for Australia, constraining to official ERPs improved the accuracy of estimates for ages 90+. The greater accuracy of constrained results compared to unconstrained results was less clear at ages 100+, however, as differences between constrained and unconstrained results were small.

SR variants were also more accurate than DG variants, indicating that estimating inputs over longer age ranges was beneficial. Differences in errors between using 3 or 5 cohorts were not significant. Furthermore, differences in errors between the SR and DG variants were greater for ages 100+ compared to younger ages. In comparison, errors varied little between different DG variants and between different SR variants. WMAPEs at ages 100+ for the SR variants varied between 19.6% and 21.6% for females and between 32.2% and 35.7% for males, whilst errors for the DG variants varied between 38.8% and 44.1% for females and between 56.2% and 65.2% for males.

Table 3 shows the Mean Absolute Percentage Error (MAPE) for the methods tested for males and females aged 90+ in New Zealand. Based on MAPEs, the variant SR(5,3,85+) is the most accurate for both males and females, followed closely by SR(5,5,85+). MAPEs for the aggregated 90+ ERPs are the same as for variants constrained to 90+ ERPs, as would be expected. With the exception of one case (DG(1,3,85+) for females), variants constrained to 85+ ERPs produced lower MAPEs than those constrained to 90+ ERPs.

**Table 3:** Mean Absolute Percentage Error for males and females aged 90+ in New Zealand over 1976–1996.

	Females	Males
SR (5,3,85+)	3.3%	4.3%
SR (5,5,85+)	3.6%	4.6%
DG (1,5,85+)	4.4%	5.1%
DG (1,5,90+)	4.6%	7.8%
DG (1,3,90+)	4.6%	7.8%
SR (5,5,90+)	4.6%	7.8%
Stats NZ ERP	4.6%	7.8%
SR (5,3,90+)	4.6%	7.8%
DG (1,3,85+)	6.3%	5.2%
SR (5,3,NC)	8.9%	8.6%
SR (5,5,NC)	9.5%	8.3%
SA (5,5,NC,10)	9.7%	8.6%
DG (1,5,NC)	10.3%	11.0%
DG (1,3,NC)	13.2%	13.4%
SA (1,5,NC,10)	13.8%	15.5%

### Changes over time

#### Australia

**Fig 5:**
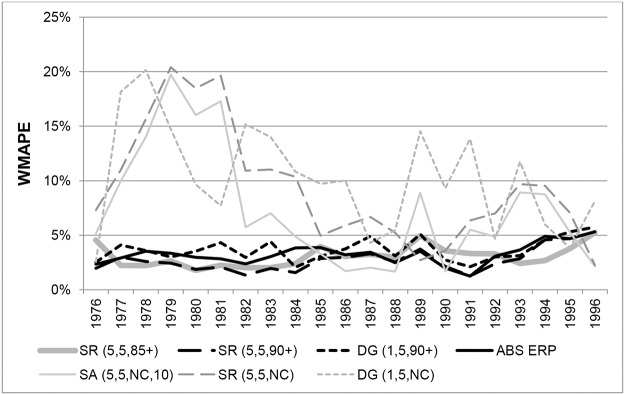
Weighted Mean Absolute Percentage Errors for Australian females aged 90+ in each year 1976–1996.

Fig 5 shows the WMAPEs for Australian females aged 90+ for different methods in each year from 1976 to 1996. Results for SR(5,3,90+) and SR(5,3,85+) are not shown because they gave very similar errors to SR(5,5,90+) and SR(5,5,85+) respectively. The ABS ERPs and the variants that produced the lowest errors on average over the whole period, SR variants constrained to ABS ERPs for ages 90+ or 85+, did so consistently throughout the period, with only a few exceptions. Unconstrained variants and the SA method generated high and volatile errors, especially between 1976 and 1981. However, the SA method was the most accurate in five of the 21 years, in 1986 to 1990 and in 1996.

**Fig 6:**
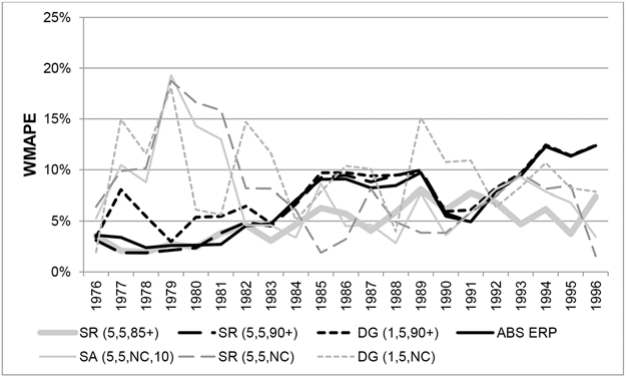
Weighted Mean Absolute Percentage Errors for Australian males aged 90+ in each year 1976–1996.

The pattern for males is similar, as can be seen in Fig 6, which shows a comparison of WMAPEs in each year from 1976 to 1996 for ages 90+. Results for SR(5,3,90+) and SR(5,3,85+) are very similar in each year to those for SR(5,5,90+) and SR(5,5,85+) and are not shown. Errors for DG variants exceeded 5% in most years and were very volatile over the period. The ABS ERPs and SR variants constrained to ERPs produced errors consistently below 5% in the years 1976 to 1982, but gradually deteriorated to 1990, producing errors of between 5% and 10%. From 1991 onwards, errors from ABS ERPs and variants constrained to 90+ ERPs consistently increased from around 5% to over 12% and were the least accurate of all the methods considered. From 1984 to 1991 the unconstrained SR variants produced the most accurate estimates, and from 1991 onwards SR(5,3,85+) and SR(5,5,85+) produced the lowest errors overall.

**Fig 7:**
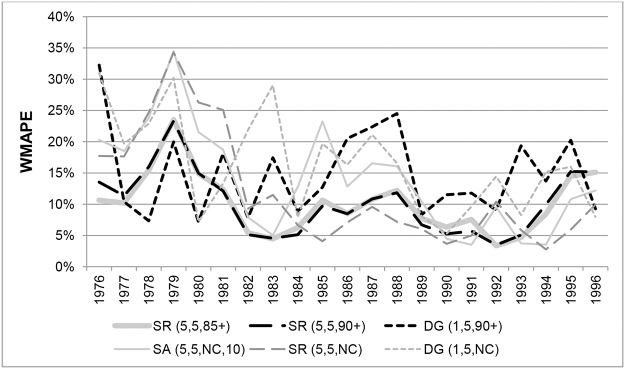
Weighted Mean Absolute Percentage Errors for Australian females aged 100+ in each year 1976–1996. Note. ABS ERPs are not included because only an aggregate ERP is available for ages 100+, which means WMAPE cannot be determined.

Fig 7 compares the WMAPEs at ages 100+ for Australian females in each year from 1976 to 1996 for the same methods as shown in Fig 5. On average over the study period, the SR method with survivor ratios measured over 5-year age ranges resulted in the lowest errors, with only marginal differences between variants which applied different ERP constraints. While all methods display volatile errors from 1976 to 1984, the SR(5,5,NC) method produced the lowest errors after 1984, and was one of the best three methods in 9 of the 12 years from 1985 to 1996.

**Fig 8:**
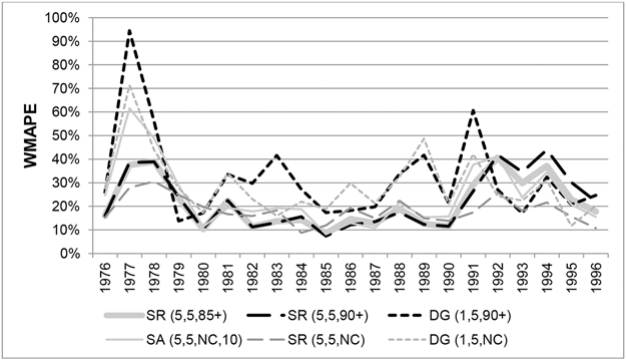
Weighted Mean Absolute Percentage Errors for Australian males aged 100+ in each year 1976–1996. Note. ABS ERPs are not included because only an aggregate ERP is available for ages 100+, which means WMAPE cannot be determined.

WMAPEs in each year at ages 100+ for Australian males are shown in Fig 8. In the period from 1980 to 1990 the SR variants and the SA(5,5,NC,10) method produced the least volatile errors, varying between 10% and 20%, but became increasing volatile and less accurate in later years. The SR(5,3,NC) and SR(5,5,NC) methods produced the lowest errors from 1991 to 1996.

**Fig 9:**
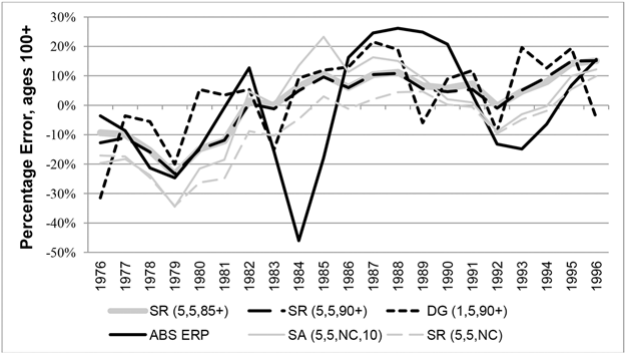
Percentage Errors at ages 100+ for Australian females in each year 1976–1996.

**Fig 10:**
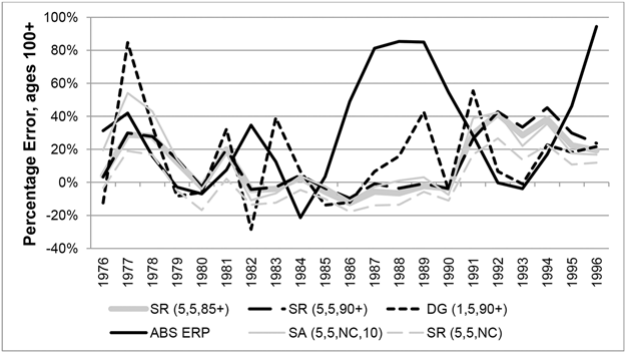
Percentage Errors at ages 100+ for Australian males in each year 1976–1996.

Percentage Errors (PEs) in years 1976 to 1996 for ABS ERPs were compared with those from variants of the SR and SA methods in Figs 9 and 10 (females and males respectively). The graphs clearly show that ABS ERPs at ages 100+ exhibit more volatile error patterns compared to methods based on nearly-extinct cohort methods. For females PEs varied between extremes of -46% in 1984 and 26% in 1988, indicating underestimation in some years and overestimation in others. For males, PEs for ERPs varied between extremes of -21% and 94%, but generally they were overestimated.

#### New Zealand

**Fig 11:**
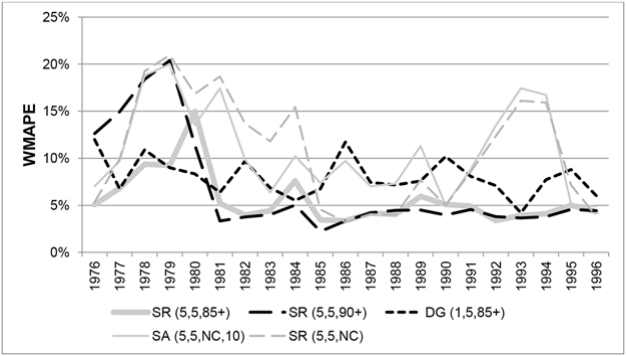
Weighted Mean Absolute Percentage Errors for females in New Zealand aged 90+ in each year 1976–1996.

Changes over time in WMAPEs for females aged 90+ are shown in Fig 11. The variants SR(5,5,85+) and SR(5,5,90+) generated high errors up to 1980 but became more accurate there-after with errors generally below 5%. From 1981 onwards SR(5,5,90+) produced the most accurate estimates at single years of age above 90, with the WMAPE varying between 2.2% and 5% in individual years. WMAPEs for SR(5,5,85+) were only marginally higher.

**Fig 12:**
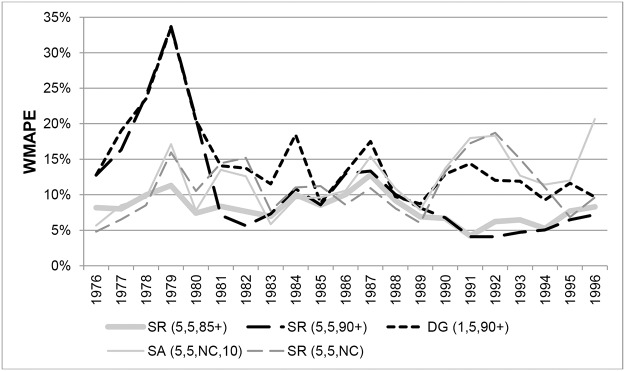
Weighted Mean Absolute Percentage Errors for males in New Zealand aged 90+ in each year 1976–1996.

WMAPEs in individual years for males aged 90+ are shown in Fig 12. The SR(5,5,85+) method produced the lowest errors on average over the whole period, followed closely by SR(5,3,85+). Following a period of large differences, the variants SR(5,5,85+) and SR(5,5,90+) produced very similar estimates from 1981. Errors from DG variants were higher and more volatile compared to SR variants, indicating that a longer age range is beneficial. Results varied very little whether survivor ratios were averaged over 3 or 5 cohorts. Errors from the unconstraint SR variant were also higher and more volatile than constrained SR variants.

**Fig 13:**
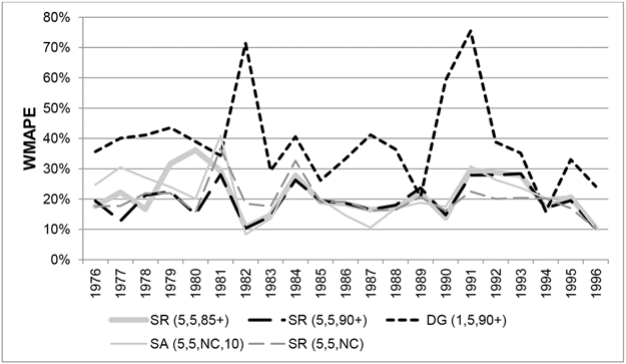
Weighted Mean Absolute Percentage Errors for females aged 100+ in New Zealand in each year 1976–1996.

Fig 13 shows the temporal variation in WMAPEs for females in New Zealand aged 100+. Amongst the DG variants, only errors for DG(1,5,90+) are shown as results for all DG variants were similar in each year. The only exceptions were DG variants based on 3 cohorts where errors increased to around 100% in 1982. With a few exceptions, errors for SR variants in each year were not hugely different whether constrained to 85+ or 90+ ERPs, or unconstrained, and also whether 3 or 5 cohorts were used.

**Fig 14:**
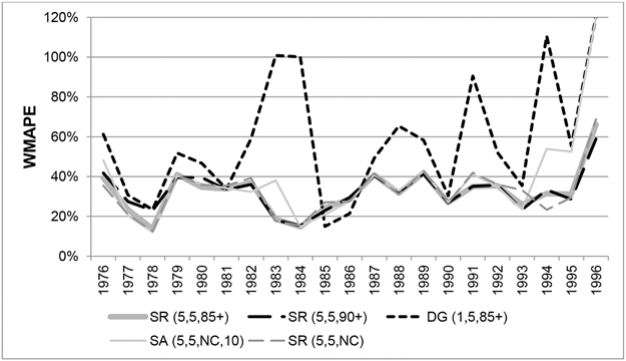
Weighted Mean Absolute Percentage Errors for males aged 100+ in New Zealand in each year 1976–1996.

WMAPEs in each year for New Zealand males aged 100+ are shown in Fig 14. Annual errors for SR(5,3,90+) and SR(5,3,NC) proved very similar to those of SR(5,3,85+). Similarly, errors for SR(5,5,90+) and SR(5,5,NC) were close to those of SR(5,5,85+). Only errors for the variants SR(5,3,85+) and SR(5,5,85+) are therefore shown. As can be seen, the SR variants produced the lowest errors, although WMAPEs varied around 30%.

**Fig 15:**
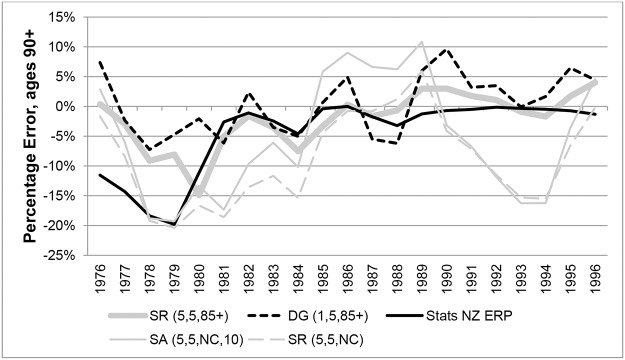
Percentage Errors at ages 90+ for females in New Zealand in each year 1976–1996.

**Fig 16:**
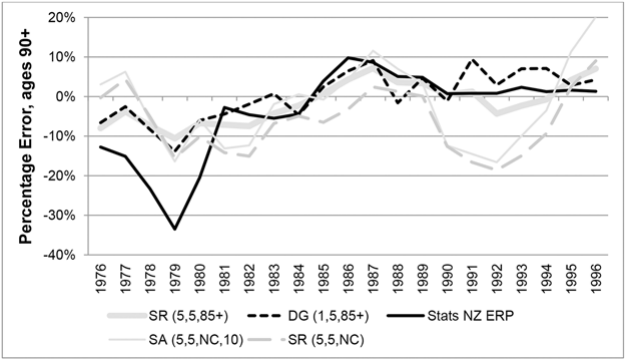
Percentage Errors at ages 90+ for males in New Zealand in each year 1976–1996.

In order to assess the accuracy of 90+ ERPs published by Statistics New Zealand, PEs in years 1976 to 1996 were calculated and are shown in Figs 15 and 16 (females and males respectively). PEs for variants constrained to 90+ ERPs were the same as those for ERPs and are thus not shown. From the graphs it is clear that from 1981 onwards, for both males and females, ERPs proved more accurate than estimates created from the SR, DG or SA methods. Prior to 1981, ERPs underestimated the total number aged 90+ by up to 20% for females and 33% for males. Thereafter ERPs slightly underestimated the number of females aged 90+ by up to only 3%. From 1985 the number of males aged 90+ was slightly overestimated by an average of 3.4%.

## Summary and Conclusions

Previous studies have evaluated alternative methods for estimating very elderly populations for a number of European countries, Japan and the US, but no evaluations had been undertaken for Australia or New Zealand. The results of one of these studies [25] were used to determine the appropriate method for estimating very elderly population numbers in the Human Mortality Database. While there are indications that official estimates for the very elderly in Australia are too high [33], no research had previously been undertaken to quantify the extent of this overestimation, and it was not known whether the methods used in the HMD, which constrain very elderly population estimates to official estimates for ages 90+, would be the most accurate for our two case study countries.

This paper assessed the accuracy of a number of population estimation methods for creating estimates at ages 85 and above based on death data over the period 1976 to 1996. The method which Thatcher, Kannisto and Andreev [25] found to be the most accurate was also shown to produce the most accurate population estimates for females aged 90+ in Australia and females aged 100+ in New Zealand. However, for Australian males aged 90+, Australian females aged 100+, New Zealand males and females aged 90+, and New Zealand males aged 100+, the SR method constrained to 85+ ERPs proved more accurate. For Australian males aged 100+, an unconstrained SR variant was most accurate. With the exception of males aged 90+ in both Australia and New Zealand, differences in the relative accuracy of SR methods constrained to 85+ ERPs and 90+ ERPs were small, however. The greater accuracy of constraining the estimates to 85+ ERPs can be partly explained by the rapid deterioration in the accuracy of ERPs with increasing age, especially for males.

With regards to the values of k (age range) and m (number of cohorts), all the results indicated that the accuracy of variants changed little whether their inputs were averaged over 3 or 5 cohorts, but that estimates were definitely more accurate when based on 5-year age ranges, as used in the SR variants, compared to one-year age ranges, as is implicit in the DG variants. The approach of adjusting results by constraining them to official 85+ or 90+ ERPs was also found to produce more accurate estimates than explicitly allowing for improvement in survival.

This paper also assessed how the various estimation methods performed over time. It was found that variants of both the SR and DG methods where no constraint was applied were prone to large variations in errors compared to estimates constrained to either 85+ or 90+ ERPs. It was more difficult to estimate populations at higher ages, as seen in the higher errors and greater volatility in errors at ages 100+ compared to ages 90+. For Australian females aged 90+, SR and DG variants where results were constrained to ABS ERPs were reasonably accurate with errors at 5% or below throughout the study period. For Australian males aged 90+, constraining to 85+ ERPs produced more accurate estimates over most of the period, especially since 1983. Considering only the period from 1985 to 1996, the most accurate methods for both Australian males and females aged 100+ proved to be SR variants unconstrained to official estimates. In New Zealand, for both males and females aged 90+ and males aged 100+, constraining to total ERPs for ages 85+ produced more accurate estimates.

Official population estimates produced by the ABS and Statistics New Zealand were mixed in their performance. ERPs produced by the ABS for ages 90+ proved reasonably reliable, although from 1991 their accuracy seems to have deteriorated. ERPs for ages 100+, in aggregate, varied significantly in accuracy from year to year. For females they alternated between substantial over- and underestimation, while for males ERPs were generally too high. On average over the study period, the accuracy of ABS ERPs for ages 100+ was poor relative to the methods evaluated, with MAPEs of 16% and 34% for females and males respectively.

In contrast, ERPs produced by Statistics New Zealand in aggregate for ages 90+ from 1981 proved very accurate, with errors mostly within 5%. On average over the study period, constraining to 85+ ERPs produced more accurate results. However, considering only the period from 1981, ERPs proved to be the most accurate.

This paper has contributed to our understanding of the performance of various nearly extinct cohort methods for estimating very elderly populations in Australia and New Zealand. It has confirmed that the SR(5,5,90+) method used in the HMD works well for Australian females aged 90 and above but that slightly more accurate estimates may be obtained for Australian males and New Zealand very elderly by constraining results from the Survivor Ratio method to ERPs for ages 85+ rather than 90+. Given that SR(5,5,90+) and SR(5,5,85+) give very similar errors for Australian females, for ease of application we would recommend the use of just SR(5,5,85+) to create very elderly population estimates for both sexes for Australia and New Zealand.

## Supporting Information

S1 Appendix Algebraic relationship between Survivor Ratio and Das Gupta methods. (DOCX)
