# Supplementary material for: An Evaluation of Nearly-Extinct Cohort Methods for Estimating the Very Elderly Populations of Australia and New Zealand
Source: PLoS One. 2015 Apr 7;10(4):e0123692. doi: 10.1371/journal.pone.0123692 (PMC4388836; doi:10.1371/journal.pone.0123692)
Supplement: S1 Appendix — (DOCX) [file pone.0123692.s001.docx]

# Appendix

In this Appendix, it is shown algebraically that population estimates derived from the survivor ratio method with an age range (k) of one year are equivalent to those estimated from applying Das Gupta’s method, provided the number of cohorts (m) in both are the same.

Survivor Ratio method with k=1:

From equation 5, the population aged x at time t can be estimated with the survivor ratio method where k=1 with:

$P_{x,t}^{c}=\frac{R_{x}}{1-R_{x}}D_{t}^{c}$ (A1)

Where, according to equation 6:

$R_{x}=\frac{\sum_{j=1}^{m} P_{x,t-j}^{c-j}}{\sum_{j=1}^{m} P_{x-1,t-1-j}^{c-j}}$ (A2)

Applying equation 3, $R_{x}$ can be re-written as:

$R_{x}=\frac{\sum_{j=1}^{m} P_{x,t-j}^{c-j}}{\sum_{j=1}^{m} P_{x,t-j}^{c-j}+D_{t-j}^{c-j}}$ (A3)

So that $\frac{R_{x}}{1-R_{x}}$ simplifies to:

$\frac{R_{x}}{1-R_{x}}=\frac{\sum_{j=1}^{m} P_{x,t-j}^{c-j}}{\sum_{j=1}^{m} D_{t-j}^{c-j}}$ (A4)

And the population aged x at time t can be written as:

$P_{x,t}^{c}=\frac{\sum_{j=1}^{m} P_{x,t-j}^{c-j}}{\sum_{j=1}^{m} D_{t-j}^{c-j}}D_{t}^{c}$ (A5)

Das Gupta’s method:

With Das Gupta’s method, the population aged x at time t is estimated with equation 1:

$P_{x,t}=\sum_{i=1}^{\omega-x} D_{t+i}^{c}$ (A6)

where (according to equation 8):

$D_{t+i}^{c}=dr_{x+i}D_{t+i-1}^{c}$ (A7)

and (equation 7):

$dr_{x+i}=\frac{\sum_{j=1}^{m} D_{t-j+i}^{c-j}}{\sum_{j=1}^{m} D_{t-j-1+i}^{c-j}}$ (A8)

Expanding equation A6:

$P_{x,t}^{c}=D_{t+1}^{c}+D_{t+2}^{c}+D_{t+3}^{c}+D_{t+4}^{c}+...$ (A9)

and re-writing it by combining it with equation A7 gives:

$P_{x,t}^{c}=D_{t}^{c}\left(dr_{x+1}+dr_{x+1}dr_{x+2}+dr_{x+1}dr_{x+2}dr_{x+3}+dr_{x+1}dr_{x+2}dr_{x+3}dr_{x+4}+...\right)$ (A10)

The numerators and denominators in consecutive death ratios (equation 7) are the same so that they cancel, simplifying this equation to:

$$P_{x,t}^{c}=D_{t}^{c}\left(\frac{\sum_{j=1}^{m} D_{t-j+1}^{c-j}}{\sum_{j=1}^{m} D_{t-j}^{c-j}}+\frac{\sum_{j=1}^{m} D_{t-j+2}^{c-j}}{\sum_{j=1}^{m} D_{t-j}^{c-j}}+\frac{\sum_{j=1}^{m} D_{t-j+3}^{c-j}}{\sum_{j=1}^{m} D_{t-j}^{c-j}}+\frac{\sum_{j=1}^{m} D_{t-j+4}^{c-j}}{\sum_{j=1}^{m} D_{t-j}^{c-j}}+...\right)$$

which simplifies to:

$P_{x,t}^{c}=\frac{D_{t}^{c}}{\sum_{j=1}^{m} D_{t-j}^{c-j}}\sum_{j=1}^{m} \sum_{i=1}^{\omega-x} D_{t-j+i}^{c-j}$ (A12)

and given that $P_{x,t-j}^{c-j}=\sum_{i=1}^{\omega-x} D_{t-j+i}^{c-j}$ this equation can be written as:

$P_{x,t}^{c}=\frac{D_{t}^{c}}{\sum_{j=1}^{m} D_{t-j}^{c-j}}\sum_{j=1}^{m} P_{x,t-j}^{c-j}$ (A13)

Or:

$P_{x,t}^{c}=\frac{\sum_{j=1}^{m} P_{x,t-j}^{c-j}}{\sum_{j=1}^{m} D_{t-j}^{c-j}}D_{t}^{c}$ (A14)

and this is the same as equation A5. This therefore shows that the survivor ratio with k=1 is equivalent to Das Gupta’s method for the same m.
